# HIV-associated lymphoproliferative disorders: Single-center experience

**DOI:** 10.1097/MD.0000000000047992

**Published:** 2026-03-06

**Authors:** Derya Demirtas, Tayfur Toptas, Asu Fergun Yilmaz, Buket Erturk Sengel, Tugba Yuksel, Yunus Emre Kokkaya, Ozlem Candan, Ahmet Mert Yanik, Secil Salim, Meral Ulukoylu Menguc, Fatma Arikan, Isik Atagunduz, Volkan Korten, Ayse Tulin Tuglular

**Affiliations:** a Division of Hematology, University of Marmara, Istanbul, Turkey; b Department of Infectious Diseases and Clinical Microbiology, University of Marmara, İstanbul, Turkey; c Department of Internal Medicine, University of Marmara, Istanbul, Turkey.

**Keywords:** acquired immune deficiency syndrome, AIDS-related lymphomas, combination antiretroviral therapy, human immunodeficiency virus, immunodeficiency, lymphoproliferative diseases

## Abstract

Survival rates of human immunodeficiency virus-positive subjects improved in recent years, especially due to innovations in current lymphoma treatments and the addition of combination antiretroviral therapy (cART). This study presents demographic and clinical characteristics, prognosis, and survival data of acquired immune deficiency syndrome (AIDS)-related lymphoma patients as a single-center experience. This retrospective study included 16 patients with AIDS-related lymphoma treated at our center between August 2010 and March 2022. Demographic data, clinical information, treatment details, and survival analysis of the patients were retrospectively reviewed from the relevant clinical archives. Fifteen out of 16 patients were males with a median age of 46.5 years (range, 24–71 years). The diagnoses were as follows: diffuse large B-cell lymphoma in 6, Burkitt lymphoma in 4, Hodgkin lymphoma in 3, plasmablastic lymphoma in 1, primary central nervous system lymphoma in 1, and chronic lymphocytic leukemia in 1 out of 16 patients. Fifteen patients presented with an advanced stage at diagnosis. Diagnosis of AIDS preceded the onset of lymphoma in 11 patients. The time from human immunodeficiency virus infection to diagnosis of lymphoma was 23.8 months (range, 1–185 months). All patients received cART during lymphoma treatment. The treatment response was evaluated in all patients who completed the initial treatment. Eleven patients (68.8%) are still alive with a complete response. Antitumour treatments administered in conjunction with cART have a favorable impact on survival and response rates in this aggressive group of patients.

## 1. Introduction

Human immunodeficiency virus (HIV) infection causes impaired cellular immunity, which predisposes to the development of neoplasms.^[1]^ Lymphomas represent one of the most frequent malignancies among individuals infected with HIV.^[2]^ The association between lymphomas and acquired immunodeficiency was identified in the early 1980s, at the start of the acquired immune deficiency syndrome (AIDS) epidemic.^[3]^

Lymphoma becomes a major problem as well as a significant cause of morbidity and mortality in HIV-infected patients.^[3]^ Diffuse large B-cell lymphoma (DLBCL) and Burkitt lymphoma (BL) are the most common subtypes. In contrast, primary central nervous system lymphomas (PCNSL), primary effusion lymphoma, plasmablastic lymphoma (PL), and classic Hodgkin lymphoma (HL) are far less frequent.^[4]^ Before the era of combination antiretroviral therapy (cART), the estimated relative risk of non-HL was 60- to 200-fold higher compared with the general population.^[5]^

The introduction of cART increased life expectancy in HIV-infected patients by improving performance status and immune function and reducing the incidence of AIDS-related lymphomas (ARL).^[6]^ However, lymphoma remains one of the most important cancer-related causes of death among HIV-infected individuals.^[7]^ Thus, future investigations are needed to identify the optimal treatment regimen for an individual patient based on disease stage, International Prognostic Index (IPI), immune status, performance status, and comorbidities.^[8]^

Therefore, in this study, we aimed to present the demographic and clinical characteristics, prognosis, and survival data of patients diagnosed with ARL who were followed and treated in a hematology clinic.

## 2. Materials and methods

This retrospective study included a total of 16 patients who were diagnosed with ARL from August 2010 to March 2022. The median age was 45 years. All but 1 patient was male. Demographic data, complete history, physical examination, and routine blood counts, as well as CD4+ and CD8+ T-cells in the peripheral blood at the time of diagnosis, were recorded from the hospital electronic database. All patients had biopsy-proven lymphoma and were staged before treatment, using the modified Ann Arbour staging system.^[9]^ Routine staging was performed, which included computerized tomography scans of the chest, abdomen, and pelvis or positron emission tomography/computerized tomography; lumbar puncture analysis; and bone marrow biopsies and aspirates, if indicated. All patients were HIV seropositive by enzyme-linked immunosorbent assay with confirmation by western blot. Complete response (CR), partial response (PR), progression, refractory disease, and relapse were defined according to the Lugano classification.^[10]^ IPI scores were assessed as previously described.^[11]^

## 3. Treatment regimens

Six patients diagnosed with DLBCL received either the rituximab, cyclophosphamide, doxorubicin, vincristine, prednisone regimen, or dose-adjusted etoposide, prednisone, vincristine, cyclophosphamide, doxorubicin, and rituximab (DA-EPOCH-R). The rituximab, cyclophosphamide, doxorubicin, vincristine, prednisone regimen comprised of prednisolone 40 mg/m^2^ D1–5 orally; rituximab 375 mg/m^2^ D1; cyclophosphamide 750 mg/m^2^ D1; doxorubicin 50 mg/m^2^ D1; and vincristine 1.4 mg/m^2^ capped at 2 mg D1 intravenous (i.v.), while the DA-EPOCH-R regimen included rituximab 375 mg/m^2^ D1; etoposide 50 mg/m^2^ D1–4; doxorubicin 10 mg/m^2^ D1–4; vincristine 0.4 mg/m^2^ D1–4; cyclophosphamide 750 mg/m^2^ D5 i.v.; and prednisone 60 mg/m^2^/bid orally D1–5. Filgrastim 5 μg/kg/d subcutaneously was given as indicated in the local protocol. A patient diagnosed with PL also received the DA-EPOCH-R regimen.

Four patients diagnosed with BL were treated with either DA-EPOCH-R or hyper-fractionated cyclophosphamide, vincristine, doxorubicin, dexamethasone, and rituximab. The hyper-fractionated cyclophosphamide, vincristine, doxorubicin, dexamethasone, and rituximab regimen consisted of 8 alternating courses administered every 21 days. Rituximab was given during courses 1 to 4. Odd courses (1, 3, 5, 7) consisted of hyper-fractionated cyclophosphamide 300 mg/m^2^ i.v. every 12 hours for 6 doses D1–3, vincristine 2 mg i.v. D4 and 11; doxorubicin 50 mg/m^2^ i.v. D4; and dexamethasone 40 mg daily D1–4 and 11 to 14. Even courses (2, 4, 6, 8) were methotrexate (MTX) and cytarabine (cytosine arabinoside) as follows: MTX 1 g/m^2^ i.v. over 24 hours D1 and cytosine arabinoside 3 g/m^2^ i.v. every 12 hours for 4 doses D2 and 3 with alkalization to promote excretion.

Three patients diagnosed with HL received the doxorubicin, bleomycin, vinblastine, dacarbazine regimen, which included doxorubicin 25 mg/m^2^ D1,15; bleomycin 10,000 units/m^2^ D1,15; vinblastine 6 mg/m^2^ D1,15; and dacarbazine 375 mg/m^2^ D1,15 i.v.

One patient diagnosed with PL lymphoma also received the DA-EPOCH regimen. One patient with chronic lymphocytic leukemia (CLL)/small lymphocytic lymphoma received ibrutinib. It is administered at a dose of 420 mg once daily and continued until disease progression or unacceptable toxicity. It was observed that 1 patient diagnosed with PCNSL was treated with a high-dose MTX regimen followed by autologous stem cell transplantation. cART treatment was given concomitant with chemotherapy in all patients.

## 4. Response criteria

CR was characterized by the complete disappearance of all measurable diseases lasting for a minimum of 4 weeks. PR was defined as a 50% reduction in the sum of the products of the cross-sectional diameters of known lesions. No response was determined as less than a PR or the occurrence of progression.

## 5. Statistical analysis

All analyses were carried out using SPSS 22.0 (Chicago) statistical software package. Descriptive statistics were used to describe the data. Data with skewed distribution were expressed as median (range). The survival time was estimated as the time elapsed between the treatment and death or last contact.

## 6. Results

Fifteen patients (93.8%) were male. Median age of patients was 46.5 years (range, 24–71 years). The diagnoses were DLBCL in 6 patients, BL in 4, HL in 3, PL in 1, PCNSL in 1, and CLL in 1 out of 16 patients. Fifteen patients presented with an advanced-stage disease. IPI scores of high-intermediate and high risk were seen in 9 (64%) patients. B symptoms, extranodal, CNS, and bone marrow involvement were present in 12 (75%), 11 (68.8%), 3 (18.8%), and 7 (43.8%) patients, respectively (Table 1).

**Table 1:** Patients and disease characteristics.

	Patients diagnosed with ARL (*n* = 16)
Age (yr)	
Median (min–max)	46.5 (24–71)
Gender (male), n (%)	15 (93.8)
Diagnosis, n (%)	
DLBCL	6 (37.5)
BL	4 (25)
HL	3 (18.8)
PL	1 (6.3)
PCNSL	1 (6.3)
CLL	1 (6.3)
Advanced stage, n (%)	15 (93.8)
Extranodal invasion, n (%)	11 (68.8)
CNS invasion, n (%)	3 (18.8)
BM invasion, n (%)	7 (43.8)
“B” symptoms, n (%)	12 (75)
HBV coinfection, n (%)	–
HCV coinfection n, (%)	1 (6.3)
CD4 + cell count, median (IQR)	210 (38–270)
HIV RNA count at diagnosis (copies/mL), median (IQR)	89,611 (40–1,870,003)
Number of patients diagnosed with lymphoma and AIDS, n (%)	5 (32)
Time from HIV infection to lymphoma diagnosis (months), median (IQR, min–max)*	23.8 (1–185)

ARL = acquired immune deficiency syndrome-related lymphoma, BL = Burkitt lymphoma, BM = bone marrow, CLL = chronic lymphocytic leukemia, DLBCL = diffuse large cell lymphoma, HBV = hepatitis B virus, HCV = hepatitis C virus, HL = Hodgkin lymphoma, PCNSL = primary central nervous system lymphoma, PL = plasmablastic lymphoma.

* Patients diagnosed with AIDS simultaneously with lymphoma were excluded.

Median CD4+ cell count and viral load at diagnosis of lymphoma were 210 cells/mL (range, 38–270 cells/mL) and 89,611 copies/mL (range, 40–1,870,003 copies/mL), respectively. Five patients (32%) were diagnosed with AIDS. The diagnosis of AIDS preceded the onset of lymphoma in 11 patients (68.8%). AIDS was diagnosed in 4 cases with *Pneumocystis jirovecii* pneumonia, 3 cases with esophageal candida infection, 1 case with *Mycobacterium tuberculosis* infection, and the other 3 patients with cytomegalovirus infection. Time from HIV diagnosis to diagnosis of lymphoma was 23.8 months (range, 1–185 months).

All patients received cART along with lymphoma treatment. cART consisted of 2 analogue nucleoside reverse transcriptase inhibitors plus 1 integrase inhibitor in 10 patients (62.5%) and 2 nucleoside reverse transcriptase inhibitors plus 1 protease inhibitor in 6 (37.5%) patients. One patient (6.3%) had a hepatitis C virus coinfection.

**Figure 1.**
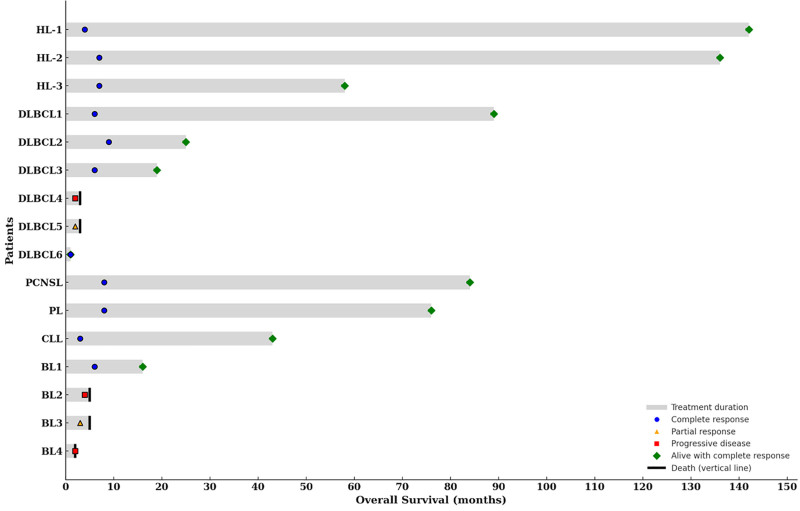
The Swimmer plot demonstrates treatment duration and overall survival in patients with HIV-associated lymphoproliferative disorders, stratified by histologic subtype (HL, DLBCL, PCNSL, PL, CLL, BL). Each horizontal bar represents an individual patient. Symbols indicate treatment response and outcomes: gray bar = treatment duration; blue circle = complete response; yellow triangle = partial response; red square = progressive disease; green diamond = alive with complete response; black vertical line = death.

Treatment response was evaluated in all patients who completed the initial treatment. Eleven patients (68.8%) achieved a CR, and 2 (12.5%) PRs; however, 3 (18.8%) experienced a progressive disease (Fig. 1). Eleven patients, who achieved a CR, had an undetectable viral load at the end of lymphoma treatment.

Individual clinical characteristics, treatment regimens, and responses of all patients are summarized in Table 2. Patients with HL uniformly achieved complete remission following doxorubicin, bleomycin, vinblastine, and dacarbazine, with durable long-term survival. In contrast, patients with aggressive non-HL subtypes, particularly BL and advanced-stage DLBCL, showed more heterogeneous outcomes, with higher rates of progression and infection-related mortality, especially among those with advanced immunosuppression.

**Table 2:** Individual clinical characteristics, treatment regimens, and responses of patients with HIV-associated lymphoproliferative disorders.

Patient No	Age	Sex	Diagnosis	Stage	Treatment	Response	Follow-up (mo)
1	48	M	HL	Advanced	ABVD	CR	142
2	36	M	HL	Advanced	ABVD	CR	140
3	32	M	HL	Advanced	ABVD	CR	58
4	50	F	DLBCL	Early	R-CHOP	CR	89
5	51	M	DLBCL	Advanced	DA-EPOCH-R	CR	24
6	59	M	DLBCL	Advanced	DA-EPOCH-R	CR	19
7	45	M	DLBCL	Advanced	R-CHOP	PD	3
8	50	M	DLBCL	Advanced	R-CHOP	PD	3
9	52	M	DLBCL	Advanced	DA-EPOCH-R	CR	3
10	25	M	PCNSL	Advanced	HD-MTX + ASCT	CR	84
11	24	M	PL	Advanced	DA-EPOCH-R	CR	76
12	41	M	CLL	Advanced	Ibrutinib	CR	43
13	60	M	BL	Advanced	DA-EPOCH-R	CR	16
14	71	M	BL	Advanced	DA-EPOCH-R	PD	5
15	36	M	BL	Advanced	Hyper-CVAD-R + HD-MTX	PD	5
16	45	M	BL	Advanced	DA-EPOCH-R	PD	3

ABVD = doxorubicin, bleomycin, vinblastine, and dacarbazine, AIDS = acquired immunodeficiency syndrome, ASCT = autologous stem cell transplantation, BL = Burkitt lymphoma, CLL = chronic lymphocytic leukemia, CR = complete response, DA-EPOCH-R = Ddose-adjusted etoposide, prednisone, vincristine, cyclophosphamide, doxorubicin, and rituximab, DLBCL = diffuse large cell lymphoma, HD-MTX = high-dose methotrexate, HIV = human immunodeficiency virus, HL = Hodgkin lymphoma, Hyper-CVAD-R = hyper-fractionated cyclophosphamide, vincristine, doxorubicin, dexamethasone, and rituximab, PCNSL = primary central nervous system lymphoma, PD = progressive disease, PL = plasmablastic lymphoma, R-CHOP = rituximab, cyclophosphamide, doxorubicin, vincristine, and prednisone.

## 7. Discussion

This report describes the demographic and clinical characteristics, prognosis, and survival of ARL patients being followed up at a single center. Our findings represent real-life experiences and provide additional insights regarding the treatment and clinical course of the ARL.

Being infected with HIV is associated with an increased risk for lymphoid malignancies as compared with the background population. The most frequent subtypes of HIV-associated lymphoid malignancies are DLBCLs and BLs, both of which have been considered as AIDS-defining illnesses since 1985.^[8]^ In our institution, from August 2010 to March 2022, we diagnosed and treated 16 patients with ARL with the following histologic types: DLBCL, n = 6 (37.5%); BL, n = 4 (25%); HL, n = 3 (18.8%); PL, n = 1 (6.3%); PCNSL, n = 1 (6.3%); and CLL, n = 1 (6.3%). This observation aligns with existing literature, emphasizing the prominence of these subtypes in the spectrum of lymphoproliferative disorders associated with HIV.

In this study, the median age at diagnosis was found to be 46.5 years, which was comparable to the global average age of the HIV-positive population.^[6]^ As advancements in cART and healthcare services continue, an aging trend is observed in the HIV-positive population. Most epidemiological studies indicate that males are more exposed to HIV-related lymphomas than females.^[12]^ The male-to-female ratio of 15:1, with a male predominance, was also observed in our study. Therefore, the age and sex distribution of HIV-associated lymphomas in our study reflects the broader context of HIV demographics worldwide.

Compared with lymphomas in the HIV-negative population, ARLs are more likely to present with advanced-stage disease, constitutional symptoms, extranodal and/or unusual location involvement.^[13]^ Fifteen out of 16 patients in our study presented with an advanced stage, typically accompanied by an abnormal LDH, B symptoms, frequent bone marrow involvement, and poor prognostic scores. In terms of clinical parameters, all patients had an aggressive disease as expected.

The immunologic profile of patients with ARL at diagnosis also changed with cART. There was an increase in median CD4+ cell count and an increase in the proportion of patients with ARL with CD4+ counts ≥ 200 cells/mm^3^. There was also a substantial decrease in HIV viral load. It appears that patients infected with HIV are developing ARL while possessing a greater CD4+ cell count and a lower HIV viral load.^[14,15]^ Despite great progress in cART and increased awareness among the population at risk, patients still tend to be diagnosed with AIDS. Those patients also had several AIDS-defining opportunistic infections at the time of diagnosis. Our observations can be translated as it seems that vulnerable groups and key populations at increased risk of HIV/AIDS still do not have a satisfactory level of awareness.

Severe immunosuppression, as measured by CD4 ≤ 200 cells/mm^3^, is a consistent predictor of poor outcomes in HIV-infected patients. In our study, death occurred in patients with CD4 ≤ 200 cells/mm^3^. This study was consistent with prior publications. Long et al^[16]^ found that, among all HIV-infected patients with cancer, low CD4 (51–200 or ≤50) was associated with a significantly increased hazard of death after controlling for other variables (2.26, 95% confidence interval = 0.98–5.23; and 4.02, 95% confidence interval = 1.75–9.26, respectively).

Concomitant cART is associated with improved CR rates and a trend toward improved overall survival in AIDS-defined lymphoma.^[17]^ The findings obtained in our study indicate that patients treated with cART along with standard chemo- or chemoimmunotherapies had high response rates and survival outcomes. In the majority of patients (68.8%), a CR was achieved and maintained for prolonged periods of time, and a decrease in HIV viral loads was also observed. However, a few patients with high viral loads and low CD4+ cell counts remained resistant to chemotherapies. This highlights the ongoing challenges and needs in the treatment of ARL. Revaluation of treatment regimens and consideration of alternative treatment options may be necessary for these patients.

This study has some important limitations. The number of patients is relatively small, and it may be better to conduct a multicenter study involving more patients. Another limitation of the study is the considerable heterogeneity of the patient profiles and the absence of a single treatment modality. Therefore, making a general assumption is challenging.

## 8. Conclusion

We conclude that the morbidity and mortality associated with HIV-related lymphomas decreased substantially with the use of cART. Patients with ARL have a near-normal response rate and survival expectancy as long as they respond to cART.

## Author contributions

**Conceptualization:** Tayfur Toptas, Asu Fergun Yilmaz, Ayse Tulin Tuglular.

**Data curation:** Derya Demirtas, Buket Erturk Sengel, Tugba Yuksel, Yunus Emre Kokkaya, Ozlem Candan, Ahmet Mert Yanik, Secil Salim, Meral Ulukoylu Menguc, Fatma Arikan.

**Formal analysis:** Derya Demirtas.

**Methodology:** Derya Demirtas, Tayfur Toptas.

**Project administration:** Derya Demirtas, Tayfur Toptas.

**Software:** Tayfur Toptas.

**Supervision:** Tayfur Toptas, Buket Erturk Sengel, Volkan Korten, Ayse Tulin Tuglular.

**Validation:** Derya Demirtas.

**Writing – original draft:** Derya Demirtas.

**Writing – review & editing:** Derya Demirtas, Tayfur Toptas, Asu Fergun Yilmaz, Isik Atagunduz, Volkan Korten, Ayse Tulin Tuglular.

## References

[1] Kimani SM Painschab MS Horner MJ. Epidemiology of haematological malignancies in people living with HIV. Lancet HIV. 2020;7:e641–51. 32791045 10.1016/S2352-3018(20)30118-1 PMC10199168

[2] de Martel C Ferlay J Franceschi S. Global burden of cancers attributable to infections in 2008: a review and synthetic analysis. Lancet Oncol. 2012;13:607–15. 22575588 10.1016/S1470-2045(12)70137-7

[3] Dunleavy K Wilson WH. How I treat HIV-associated lymphoma. Blood. 2012;119:3245–55. 22337719 10.1182/blood-2011-08-373738 PMC3321851

[4] Riedel DJ Rositch AF Redfield RR Blattner WA. HIV-associated lymphoma sub-type distribution, immunophenotypes and survival in an urban clinic population. Leuk Lymphoma. 2016Feb;57:306–12. 26025299 10.3109/10428194.2015.1055483

[5] Polesel J Clifford GM Rickenbach M; Swiss HIV Cohort Study. Non-Hodgkin lymphoma incidence in the Swiss HIV cohort study before and after highly active antiretroviral therapy. AIDS. 2008;22:301–6. 18097233 10.1097/QAD.0b013e3282f2705d

[6] Gopal S Patel MR Yanik EL. Temporal trends in presentation and survival for HIVassociated lymphoma in the antiretroviral therapy era. J Natl Cancer Inst. 2013;105:1221–9. 23892362 10.1093/jnci/djt158 PMC3748003

[7] Horner MJ Shiels MS Pfeiffer RM Engels EA. Deaths attributable to cancer in the US human immunodeficiency virus population during 2001–2015. Clin Infect Dis. 2021;72:e224–31. 32710777 10.1093/cid/ciaa1016 PMC8096269

[8] Meister A Hentrich M Wyen C Hübel K. Malignant lymphoma in the HIV-positive patient. Eur J Haematol. 2018;101:119–26. 29663523 10.1111/ejh.13082

[9] Ansell SM. Hodgkin lymphoma: 2025 update on diagnosis, risk-stratification, and management. Am J Hematol. 2024;99:2367–78. 39239794 10.1002/ajh.27470

[10] Cheson BD Fisher RI Barrington SF; Alliance, Australasian Leukaemia and Lymphoma Group. Recommendations for initial evaluation, staging, and response assessment of Hodgkin and non-Hodgkin lymphoma: the Lugano classification. J Clin Oncol. 2014;32:3059–68. 25113753 10.1200/JCO.2013.54.8800 PMC4979083

[11] Chen J Liu X Qin S. A novel prognostic score including the CD4/CD8 for AIDS-related lymphoma. Front Cell Infect Microbiol. 2022;12:919446. 35873145 10.3389/fcimb.2022.919446 PMC9299417

[12] Bohlius J. Incidence and risk factors of HIV-associated non-Hodgkin-lymophma in the era combined anti-retroviral therapy. a European multi-cohort study. Infect Agent Cancer. 2009;4:O2.

[13] Noy A. HIV lymphoma and burkitts lymphoma. Cancer J. 2020;26:260–8. 32496459 10.1097/PPO.0000000000000448 PMC9302611

[14] Panel on Guidelines for the Prevention and Treatment of Opportunistic Infections in Adults and Adolescents With HIV. Guidelines for the Prevention and Treatment of Opportunistic Infections in Adults and Adolescents With HIV. National Institutes of Health, HIV Medicine Association, and Infectious Diseases Society of America. https://clinicalinfo.hiv.gov/en/guidelines/adult-and-adolescent-opportunistic-infection. Accessed February 27, 2026.

[15] Mathoma A Sartorius B Mahomed S. The trends and risk factors of AIDS-defining cancers and non-AIDS-defining cancers in adults living with and without HIV: a narrative review. J Cancer Epidemiol. 2024;2024:7588928. 38549952 10.1155/2024/7588928 PMC10978085

[16] Long JL Engels EA Moore RD Gebo KA. Incidence and outcomes of malignancy in the HAART era in an urban cohort of HIV-infected individuals. AIDS. 2008;22:489–96. 18301061 10.1097/QAD.0b013e3282f47082 PMC2553213

[17] Barta SK Xue X Wang D. Treatment factors affecting outcomes in HIV-associated non-Hodgkin lymphomas: a pooled analysis of 1546 patients. Blood. 2013;122:3251–62. 24014242 10.1182/blood-2013-04-498964 PMC3821722

